# Supplementary material for: Addition of Vindoline to p-Benzoquinone: Regiochemistry, Stereochemistry and Symmetry Considerations
Source: Molecules. 2021 Oct 22;26(21):6395. doi: 10.3390/molecules26216395 (PMC8587549; doi:10.3390/molecules26216395)
Supplement: Supplementary file 1 [file molecules-26-06395-s001.zip › molecules-1430516-supplementary.pdf]

Supplementary Materials: Addition of vindoline to p-benzoquinone: regiochemistry, stereochemistry and symmetry considerations

Shamsher Ali, Ritchy Leroy, Eric Hénon and Georges Massiot

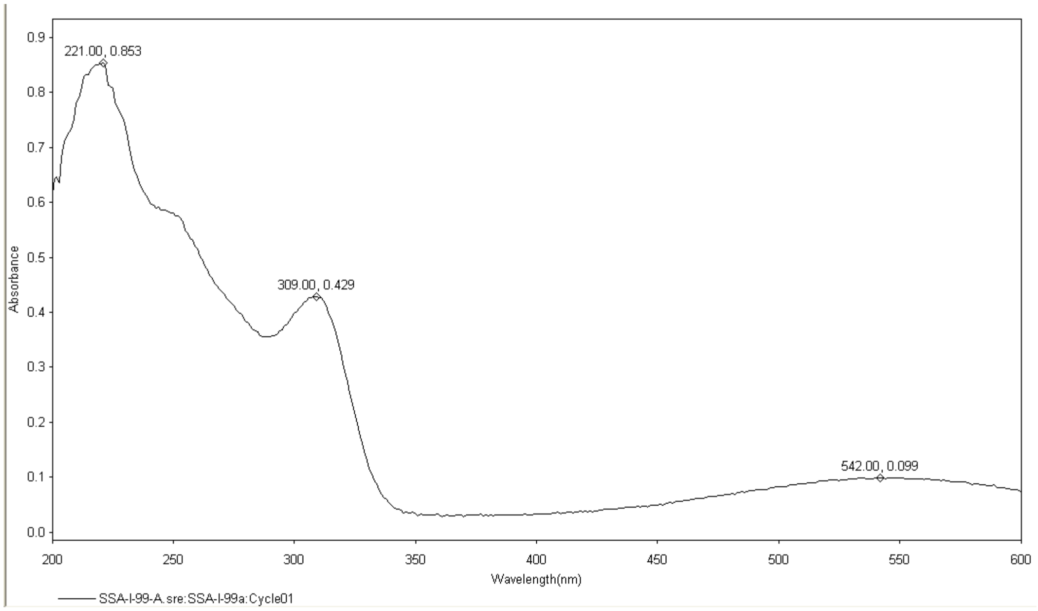

Figure S1: UV spectrum of compound **3**

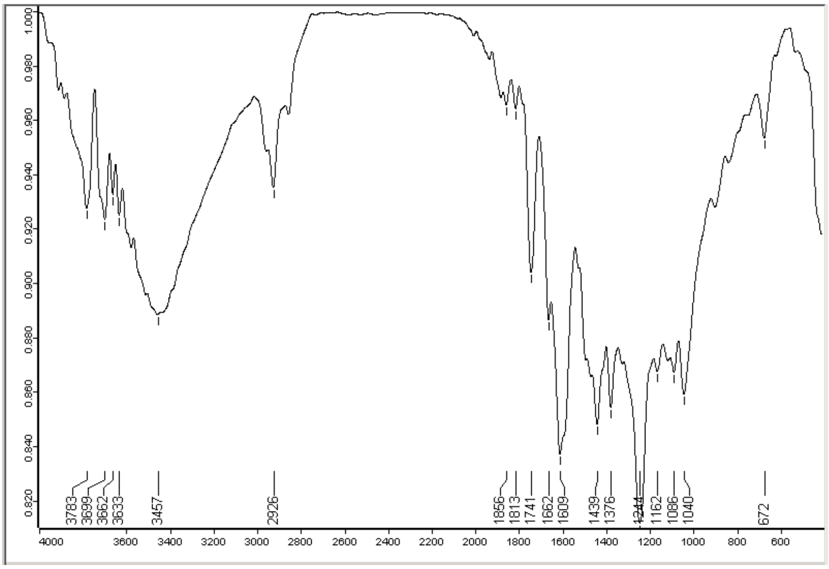

Figure S2: IR spectrum of **compound 3**

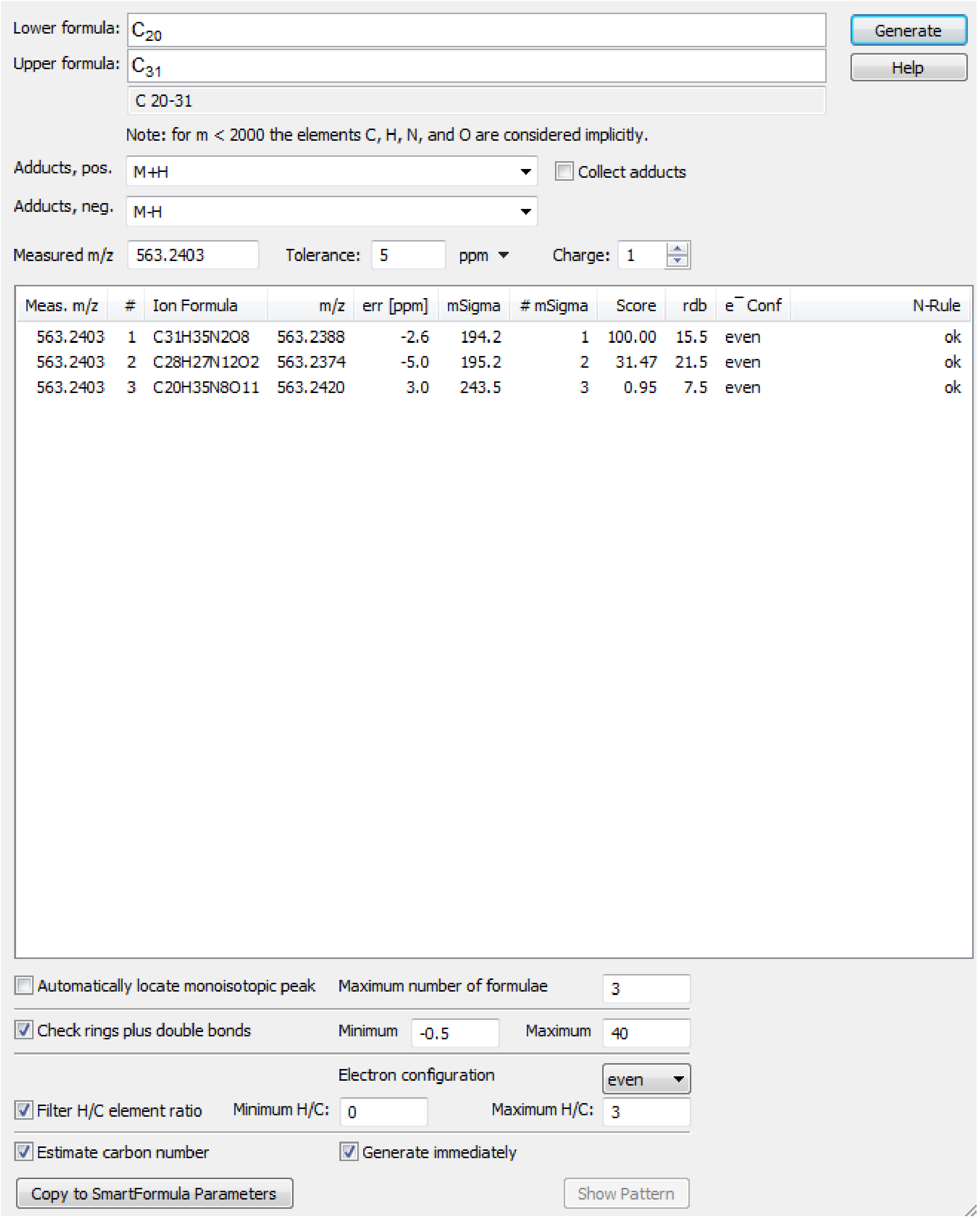

Figure S3: HRESI-MS spectrum of compound **3**

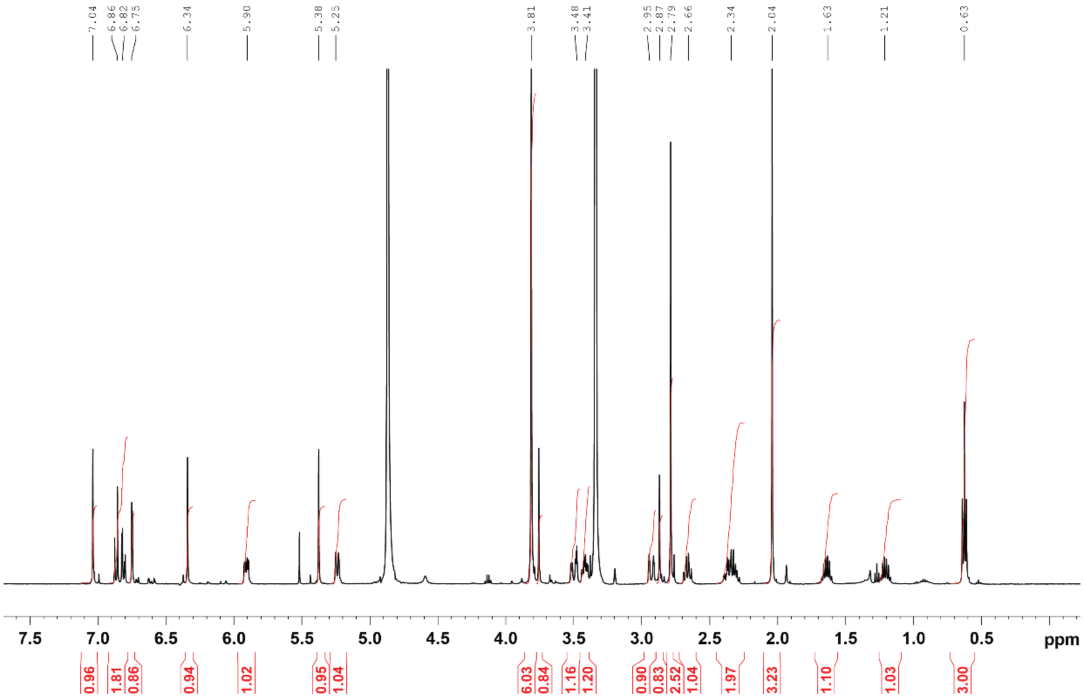

Figure S4: ^{1}H NMR spectrum of compound **3**

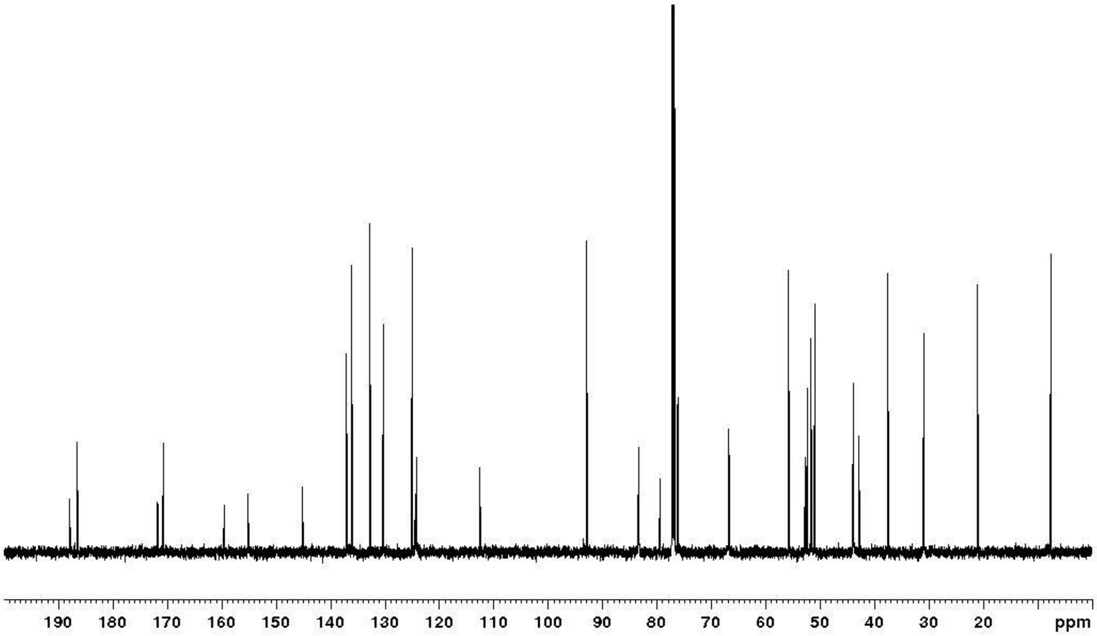

Figure S5: ^{13}C NMR spectrum of compound **3**

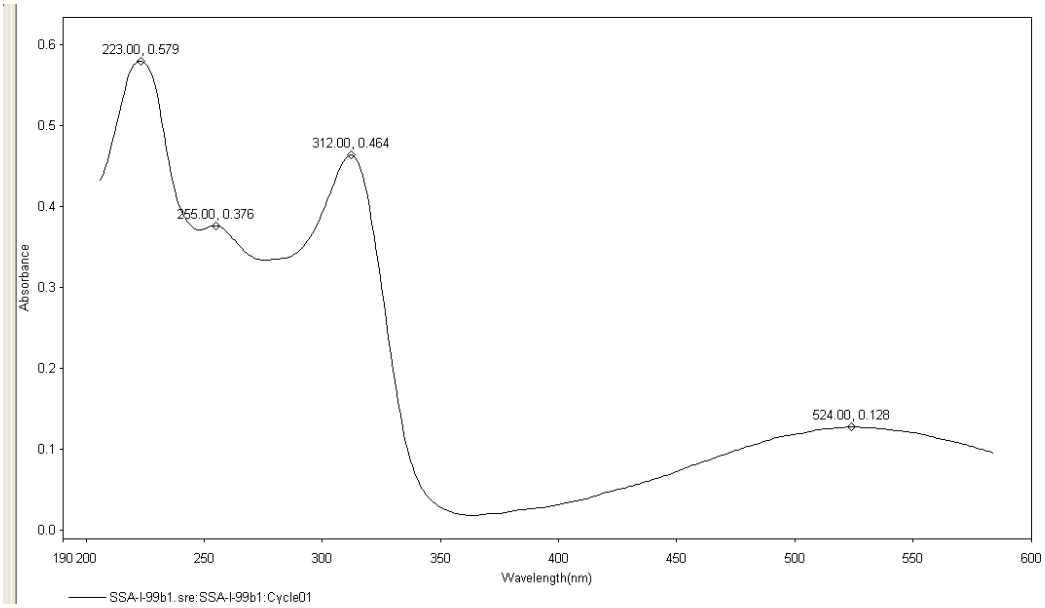

Figure S6: UV spectrum of compound **4**

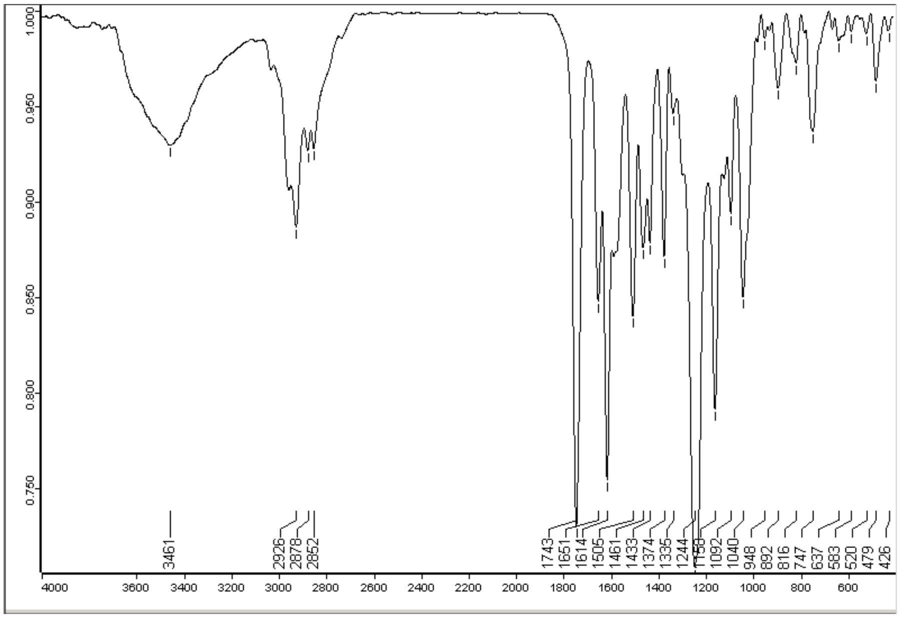

Figure S7: IR spectrum of compound 4

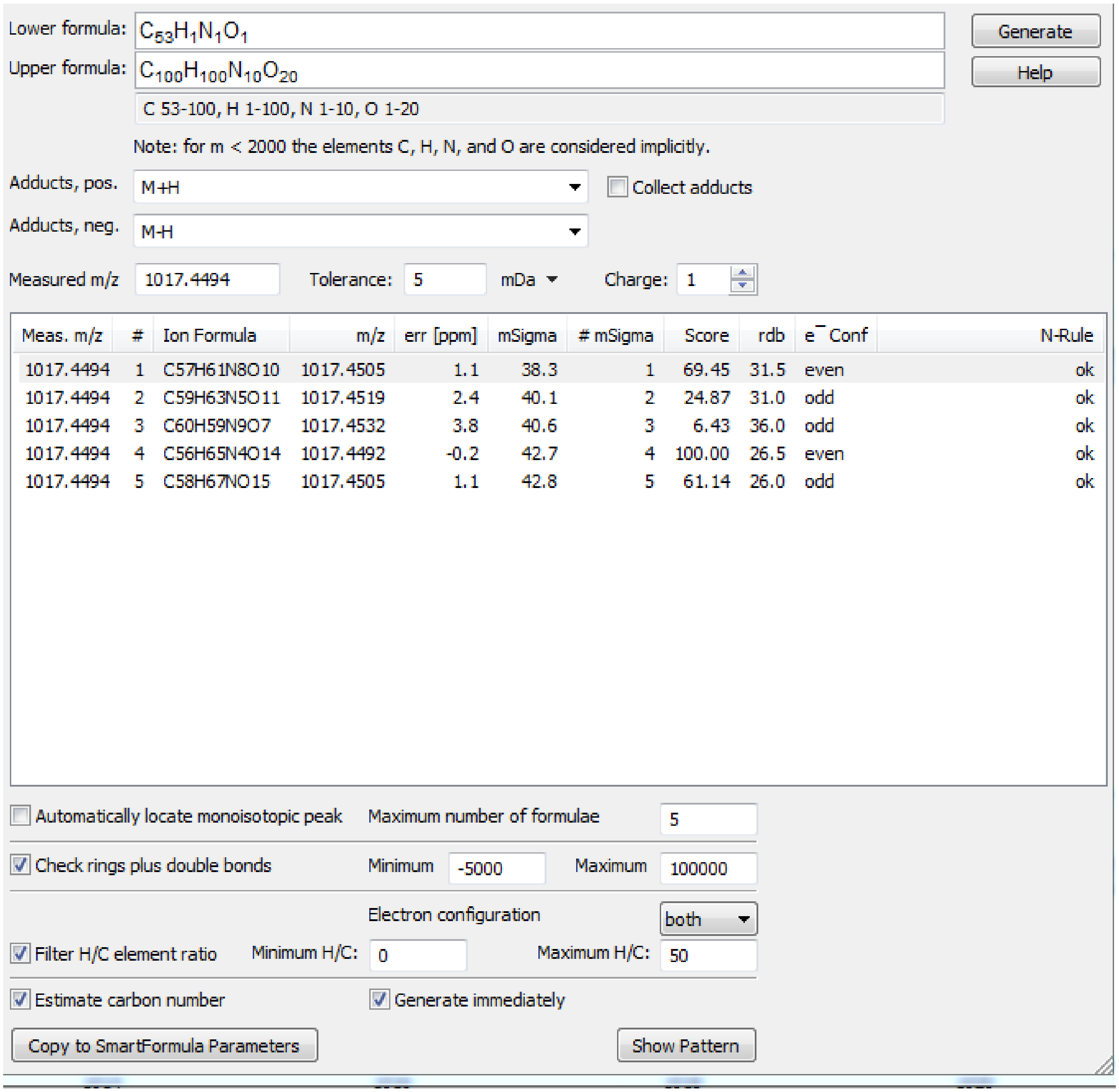

Figure S8: HRESI-MS spectrum of compound **4**

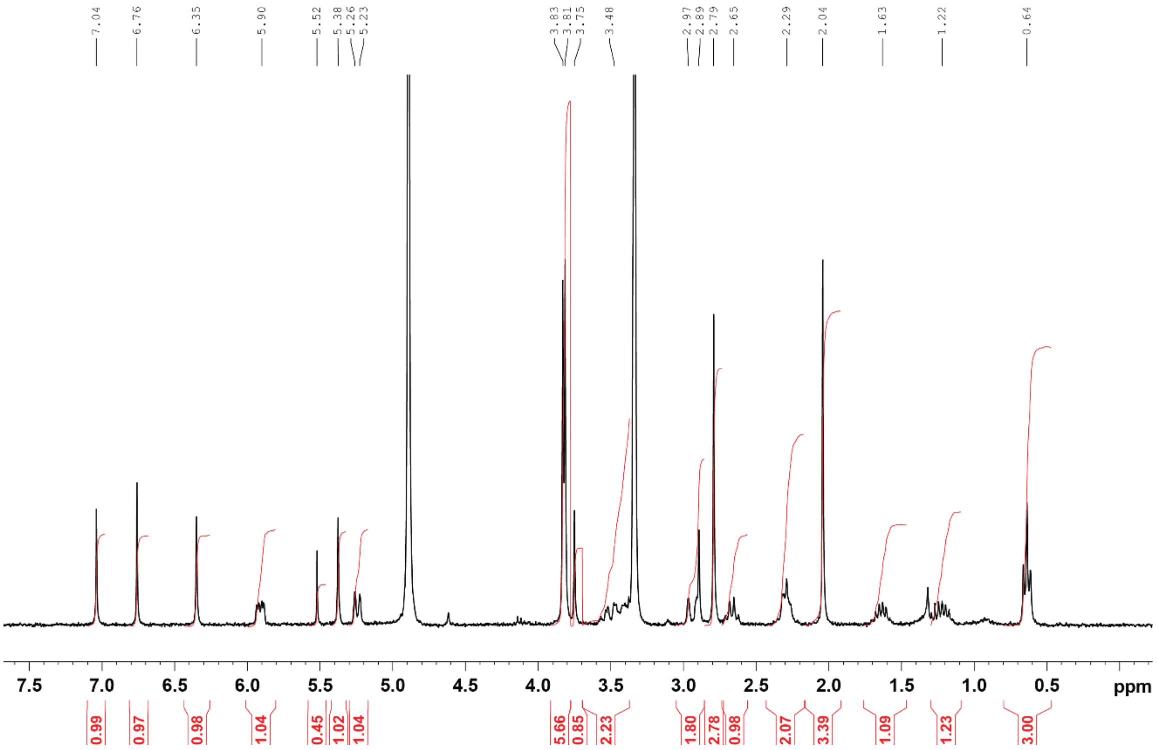

Figure S9: ^{1}H NMR spectrum of compound **4**

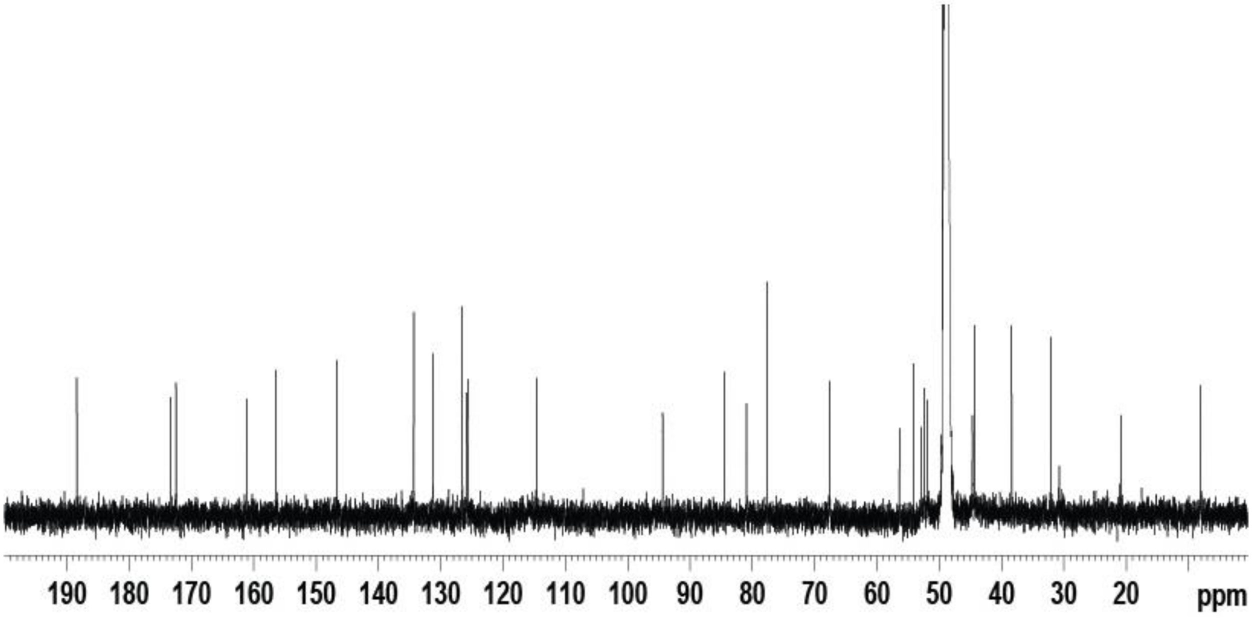

Figure S10: ^{13}C NMR spectrum of compound **4**

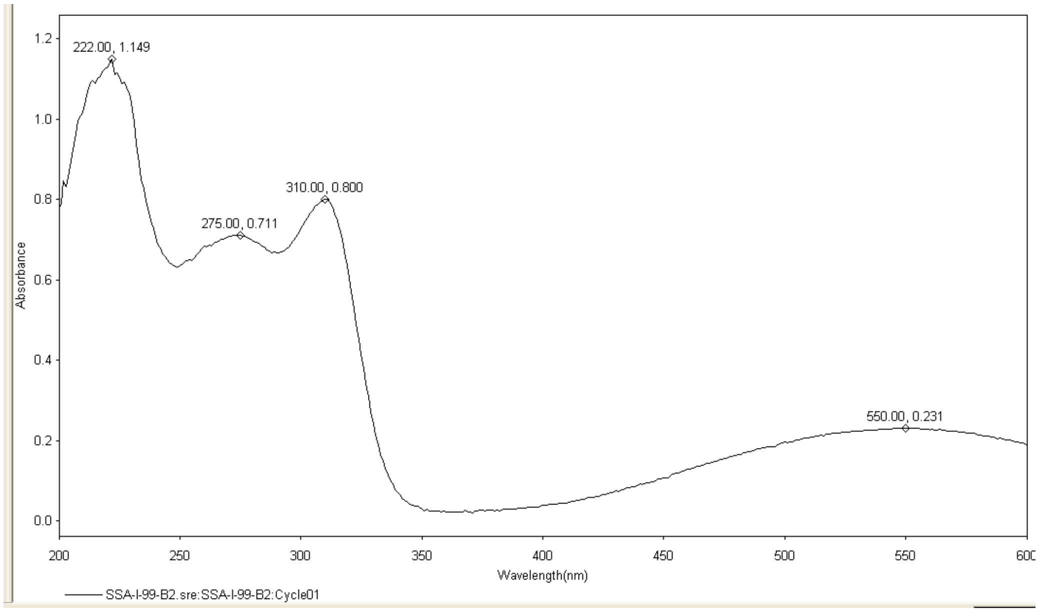

Figure S11: UV spectrum of compound **5**

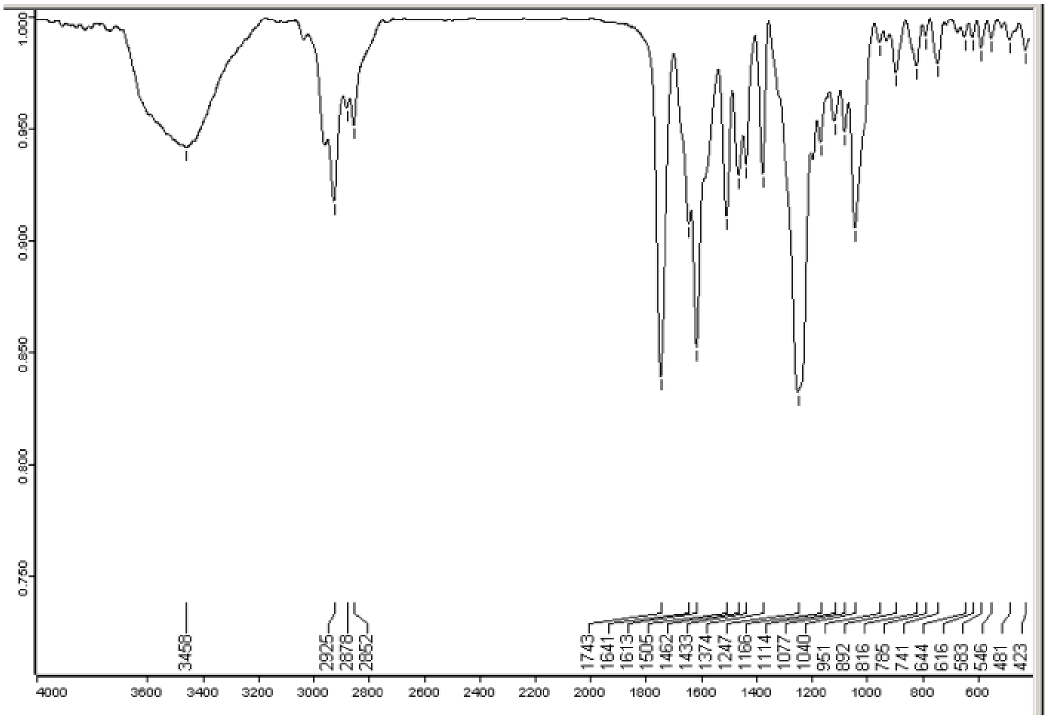

Figure S12: IR spectrum of compound **5**

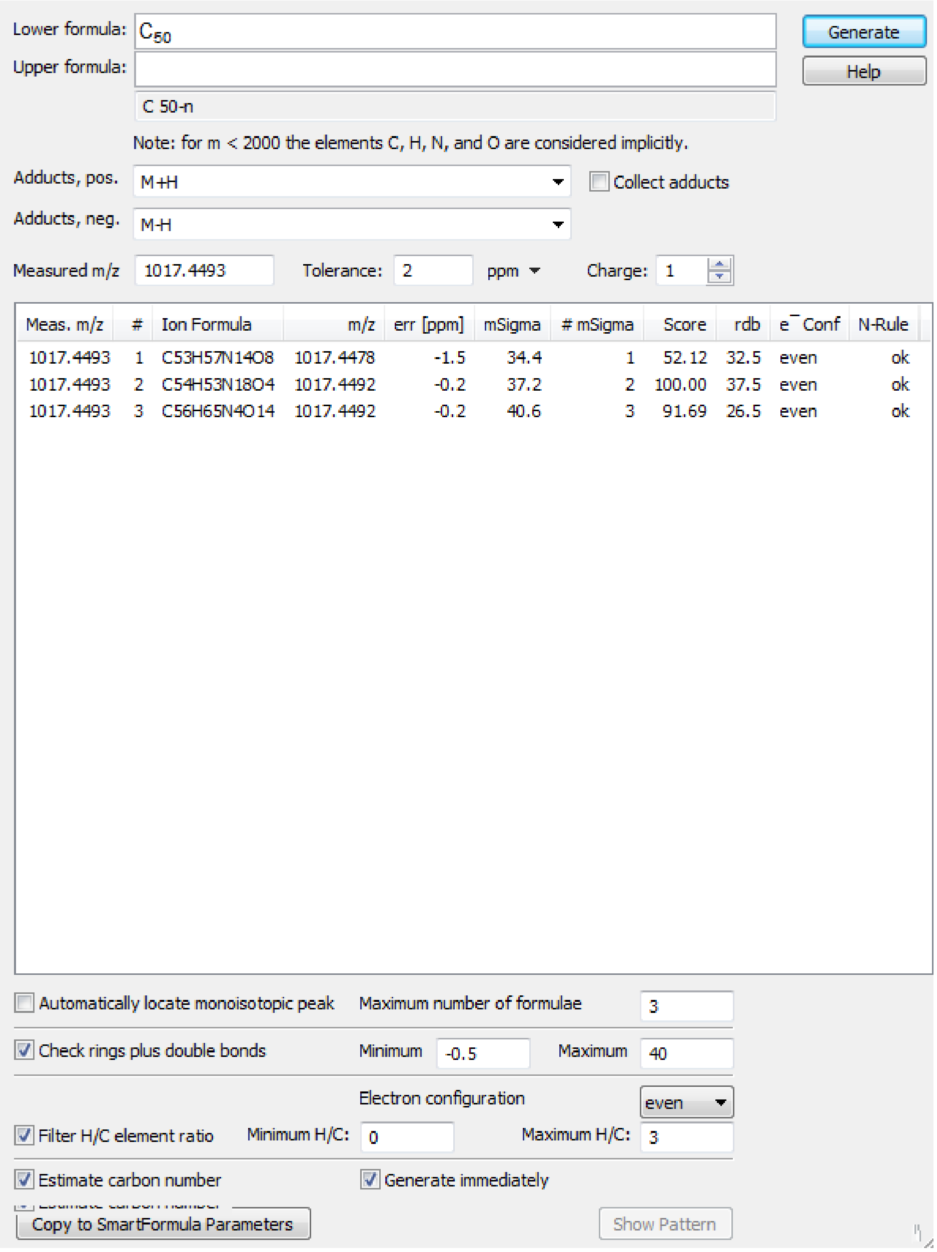

Figure S13: HRESI-MS spectrum of compound **5**

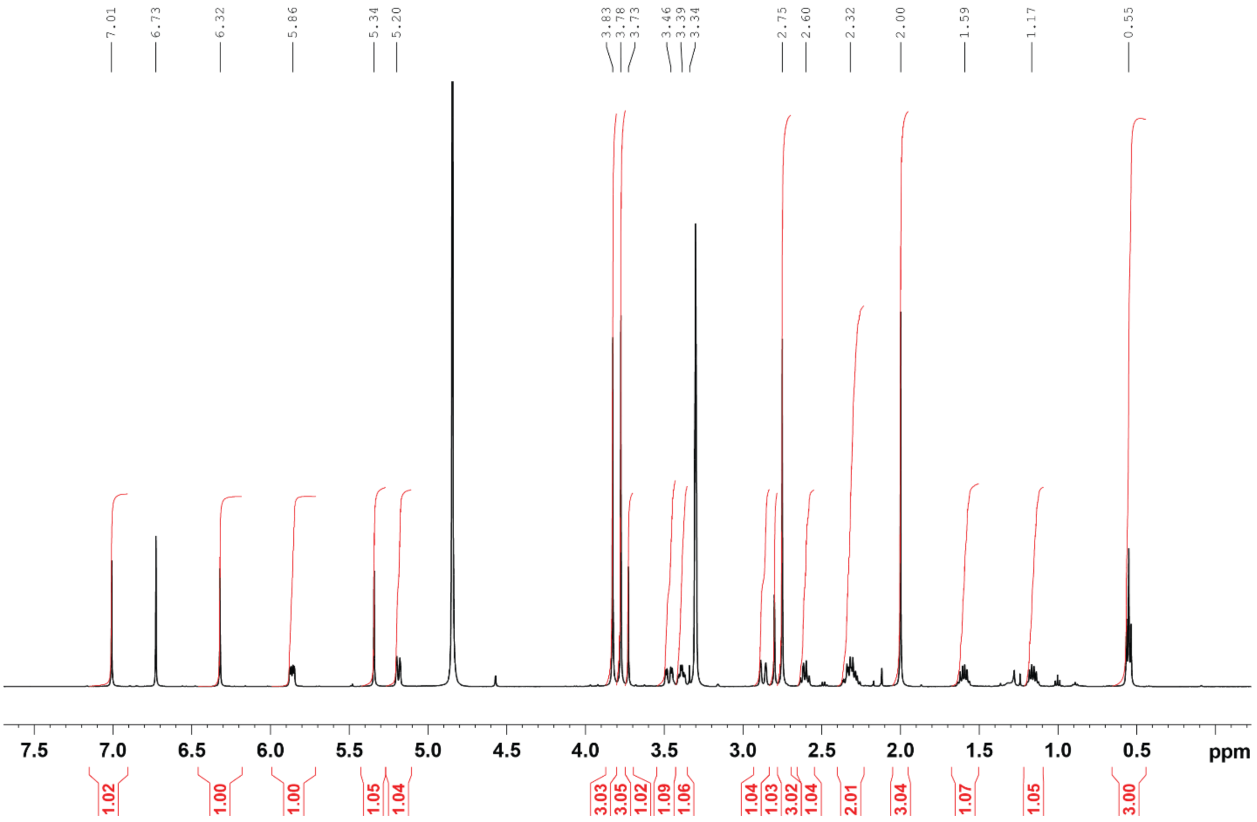

Figure S14: ^{1}H NMR spectrum of compound **5** (peak at 5.5 ppm is CH_2Cl_2).

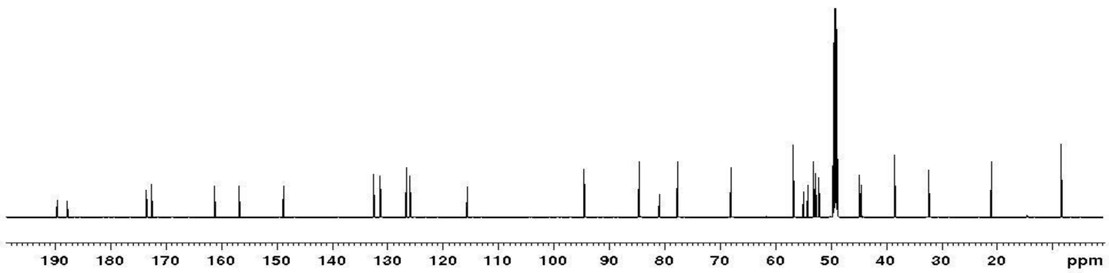

Figure S15: ^{13}C NMR spectrum of compound **5**

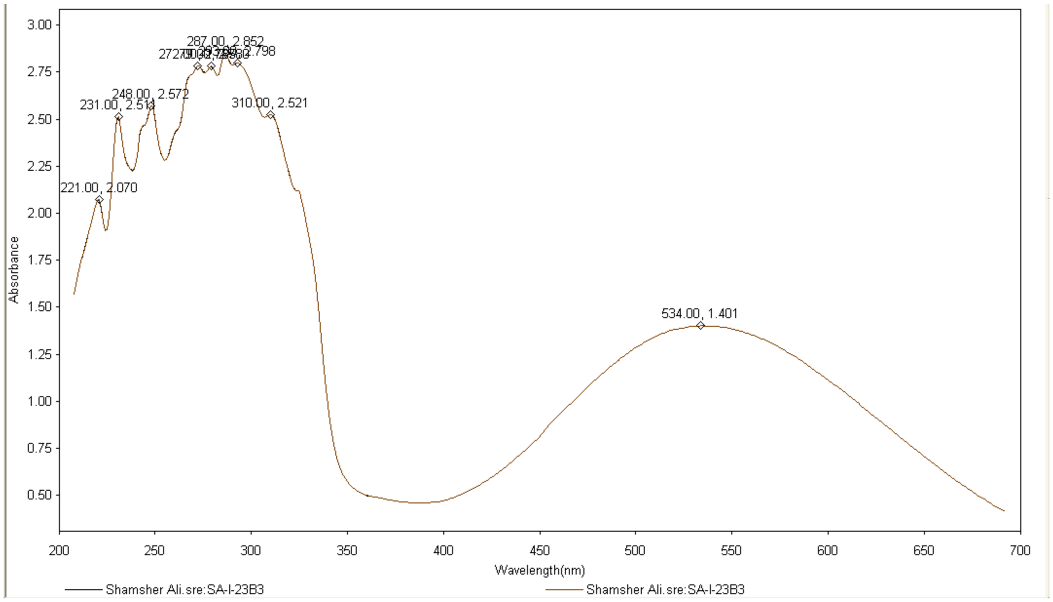

Figure-16: UV spectrum of compounds **6**

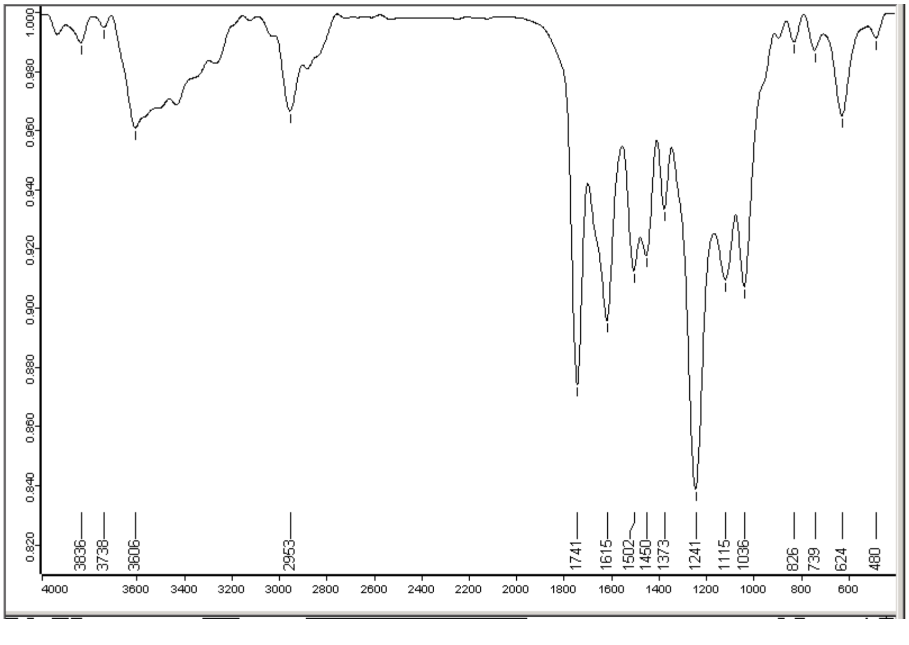

Figure S17: IR spectrum of compounds **6**

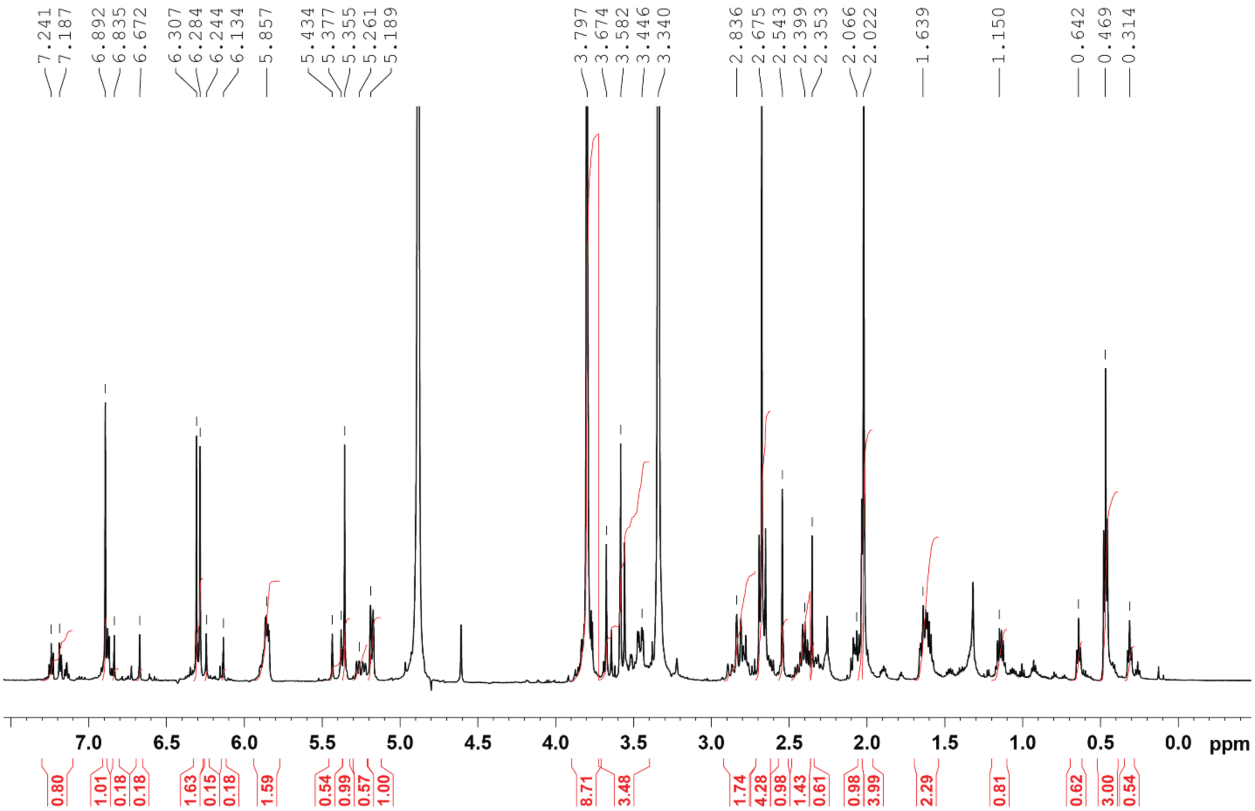

Figure S18: ^{1}H NMR spectrum of compounds **6**

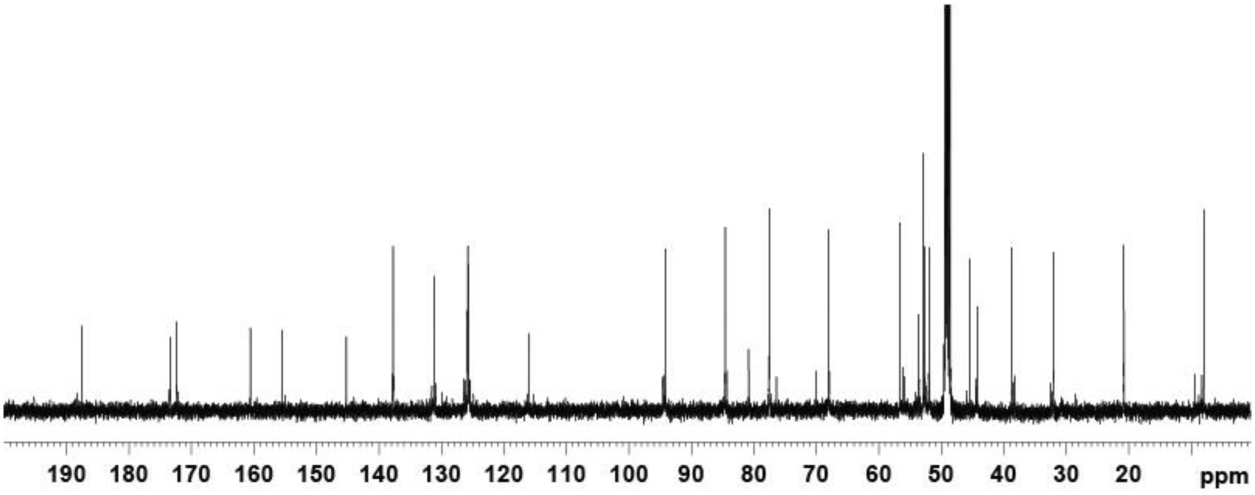

Figure S19: ^{13}C NMR spectrum of compound **6**

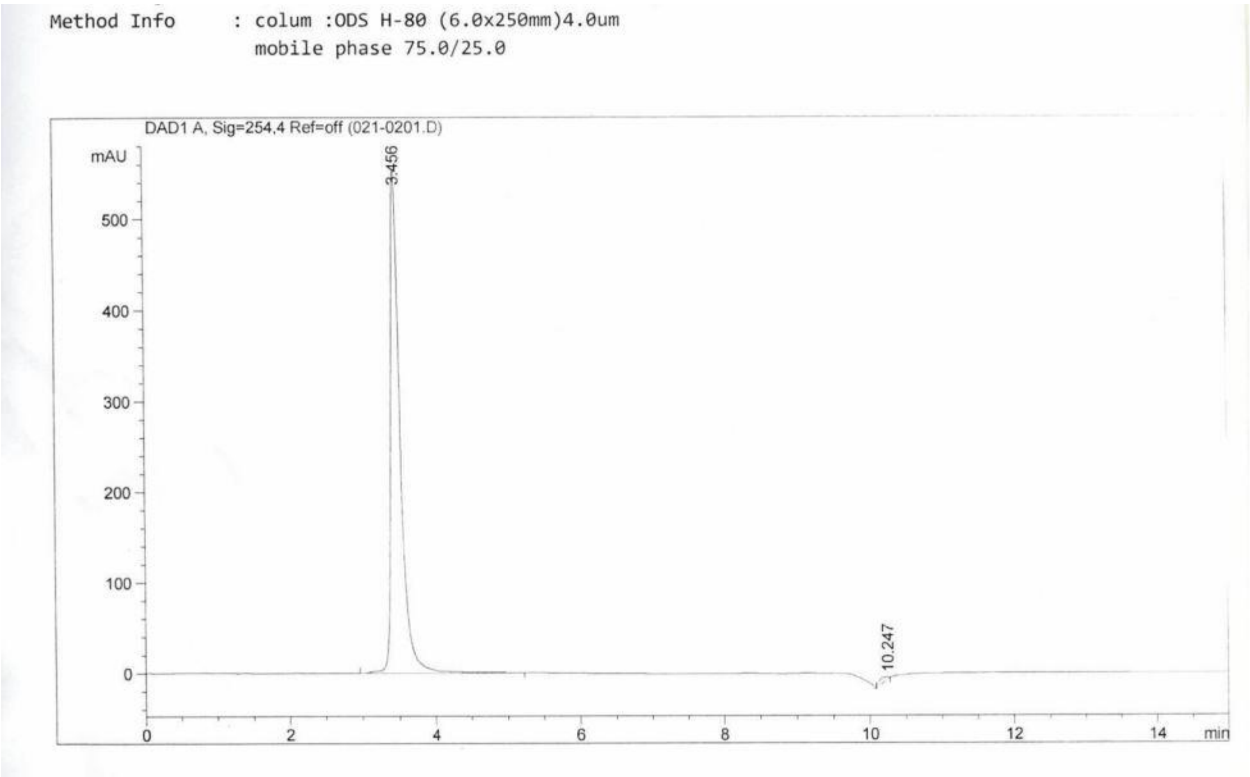

Figure S20: UPLC profile of compound **6**

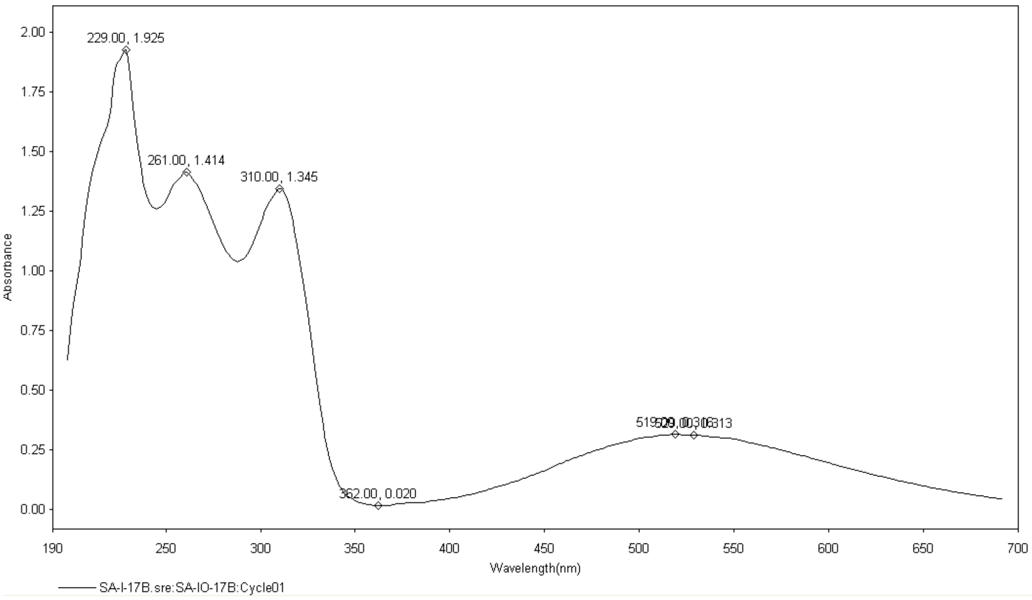

Figure S21: UV spectrum of compounds **7**

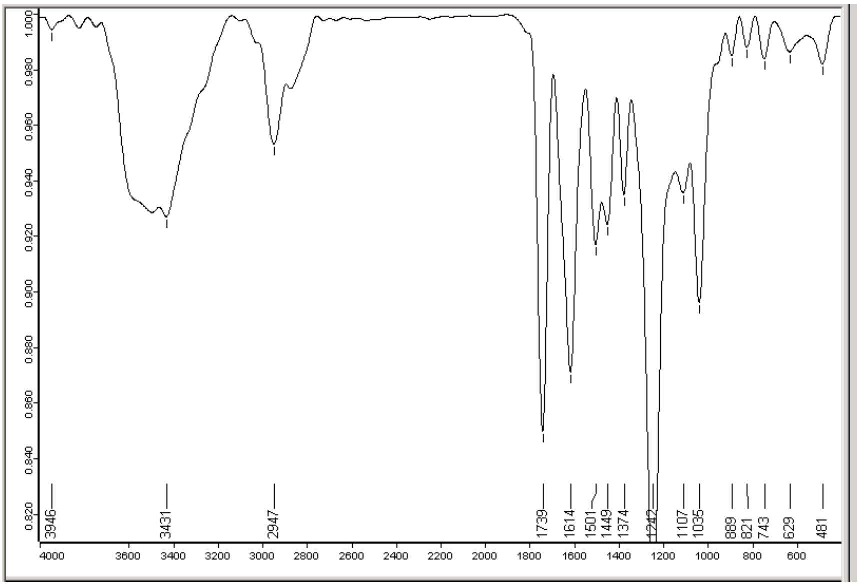

Figure S22: IR spectrum of compounds **7**

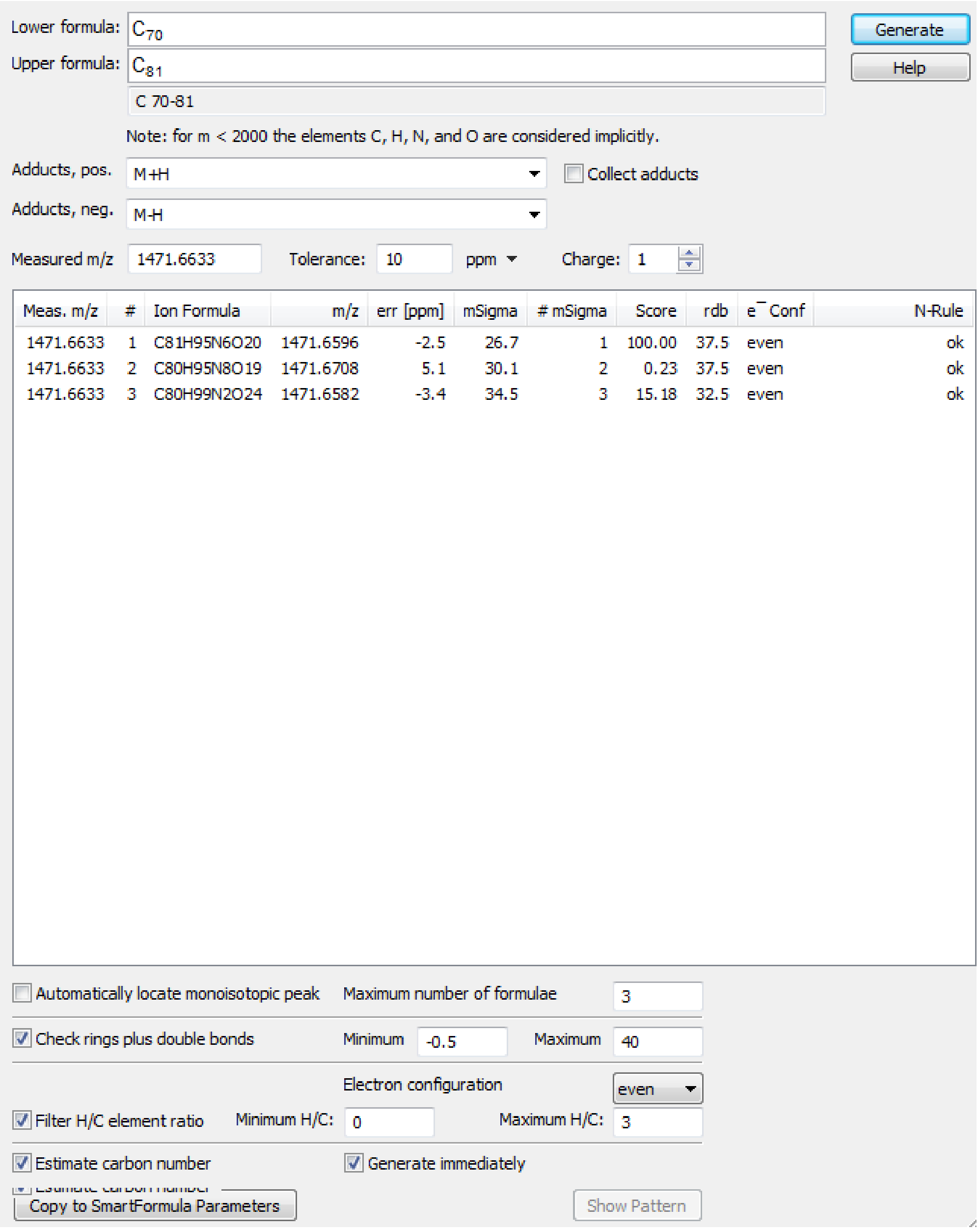

Figure S23: HRESI-MS spectrum of compounds **7**

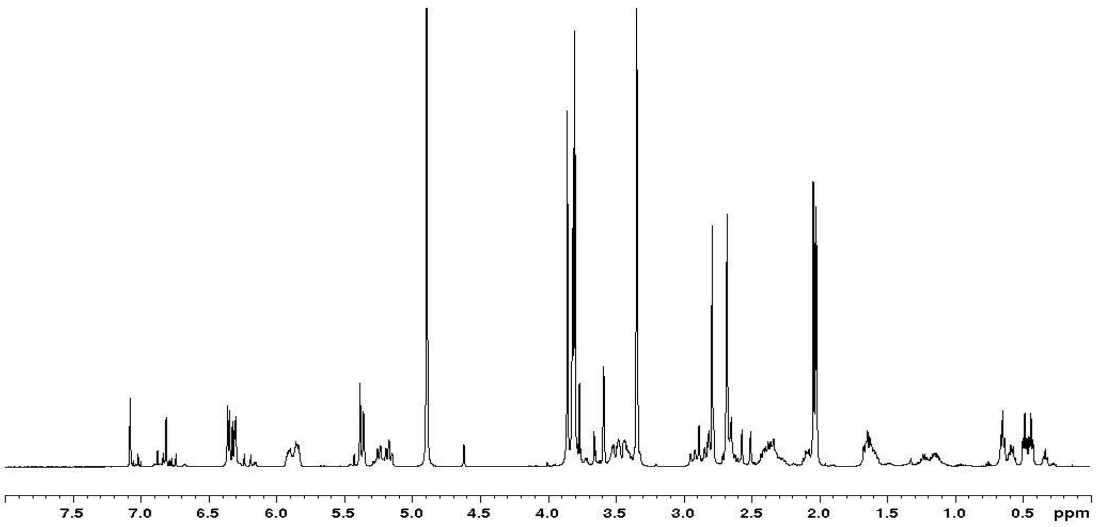

Figure S24: ^{1}H NMR spectrum of compounds **7**

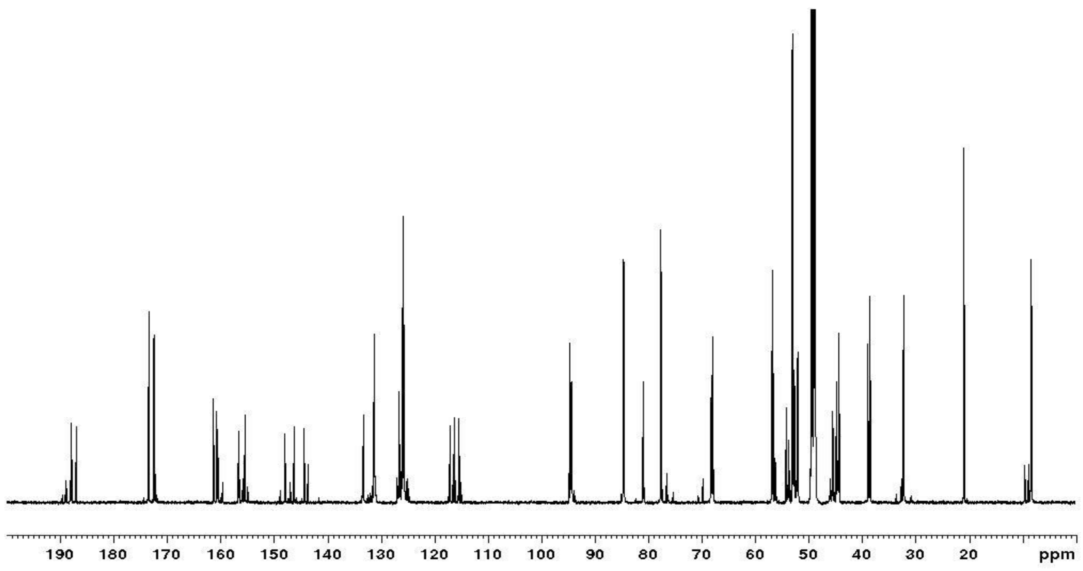

Figure S25: ^{13}C NMR spectrum of compounds **7**

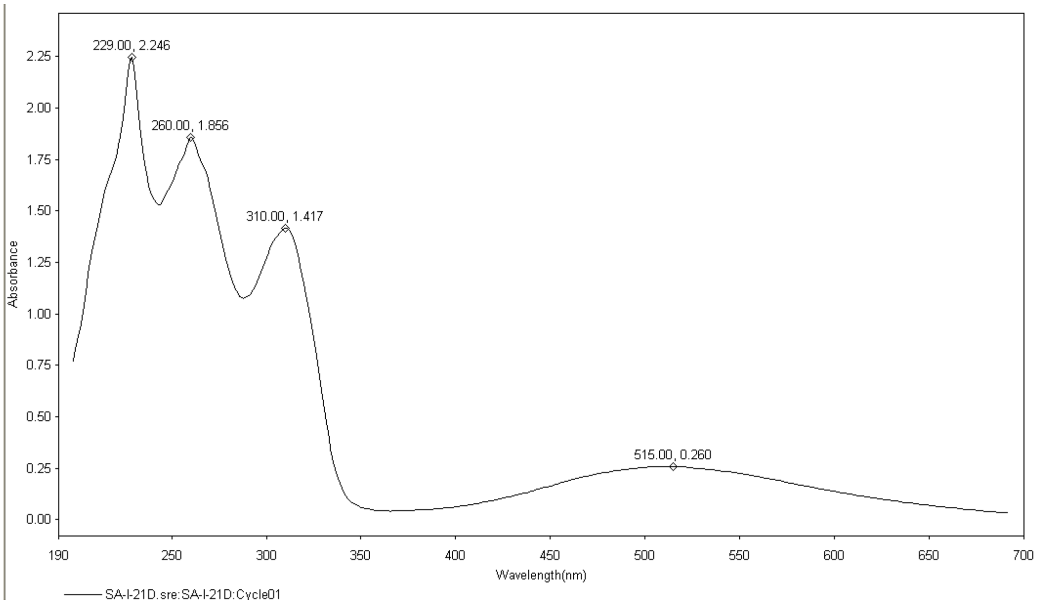

Figure S26: UV spectrum of compounds **8**

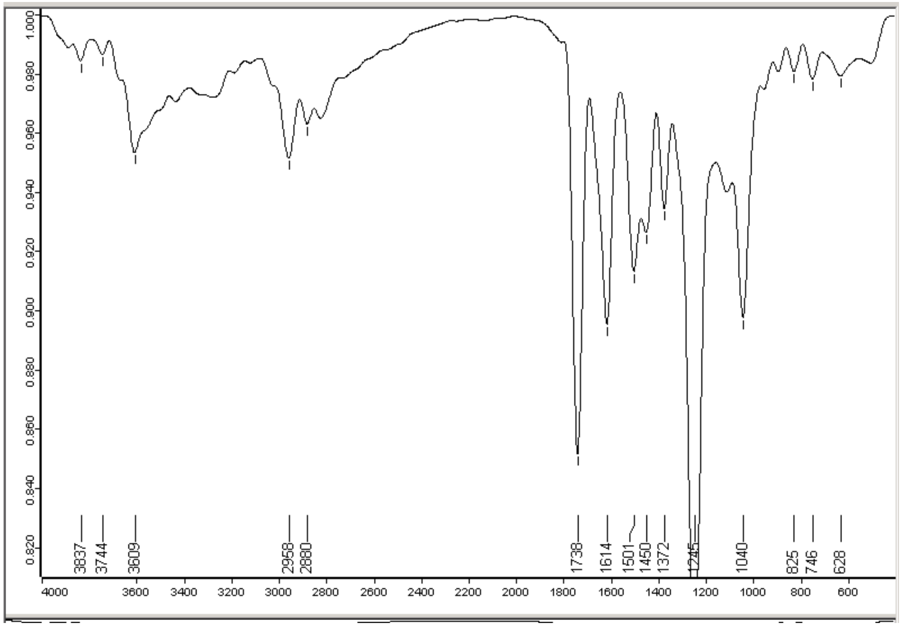

Figure S27: IR spectrum of compounds **8**

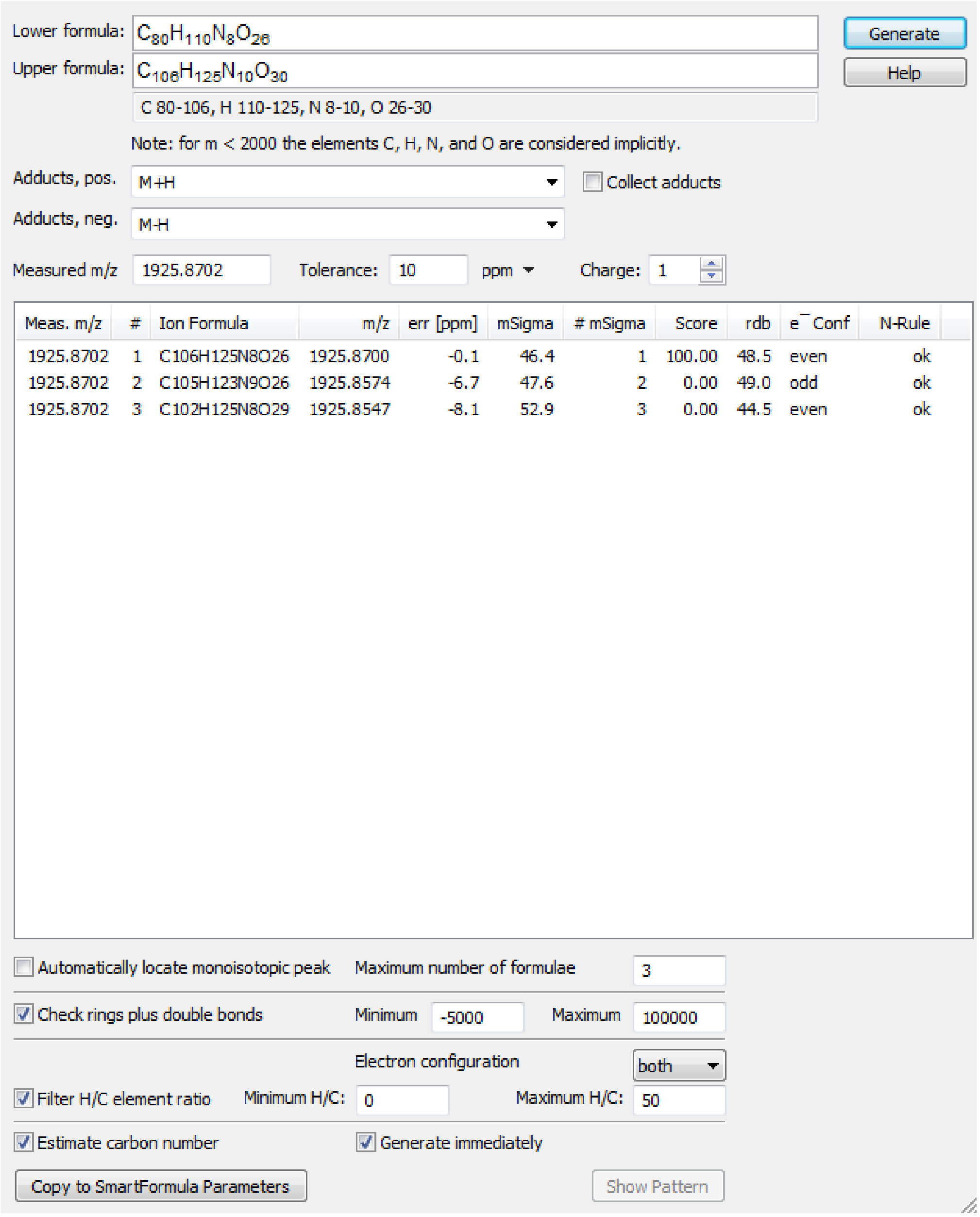

Figure S28: HR-ESI-MS spectrum of compounds **8**

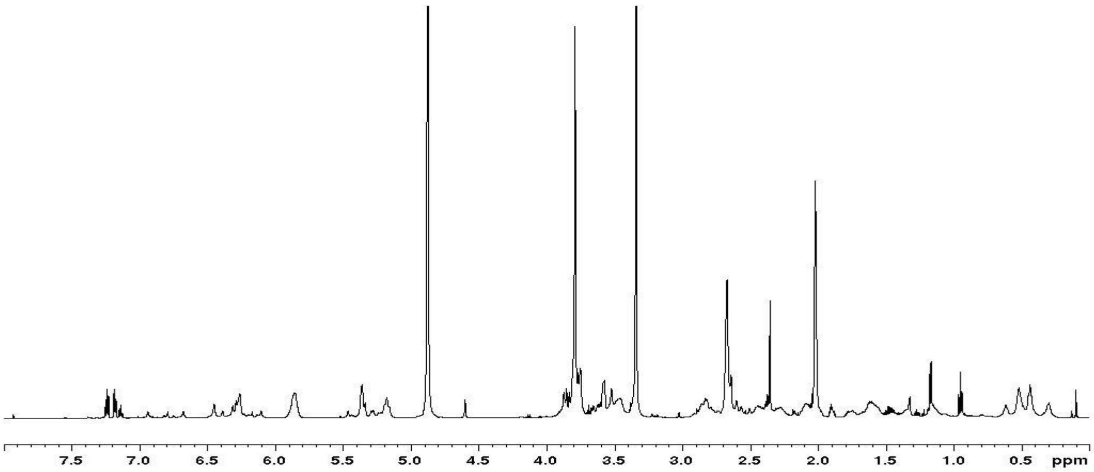

Figure S29: ^{1}H NMR spectrum of compounds **8**

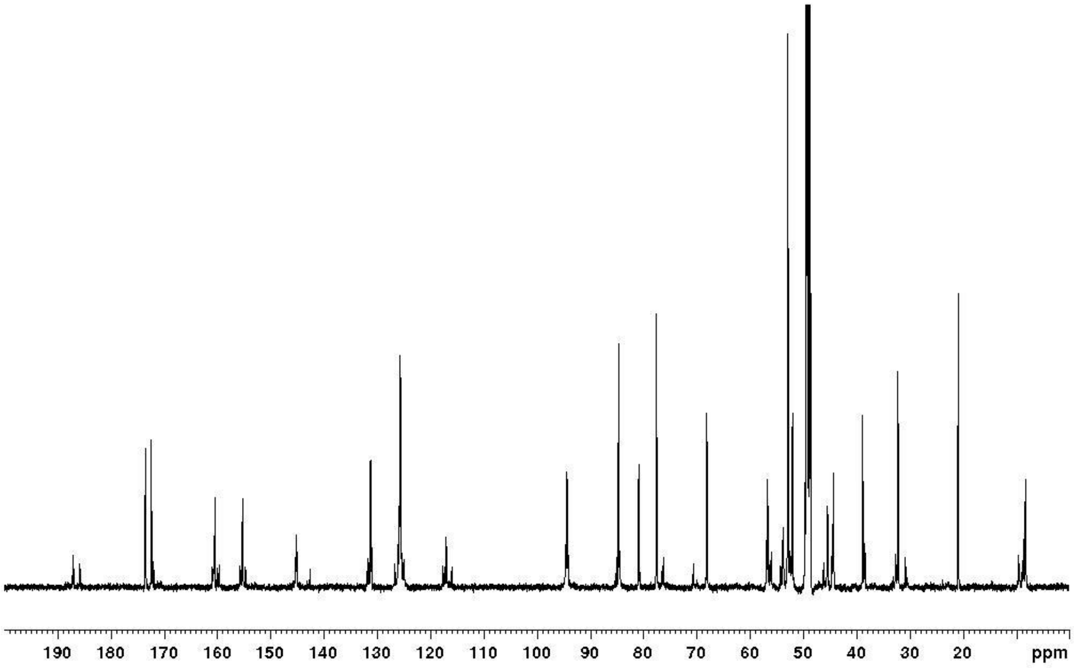

Figure S30: ^{13}C NMR spectrum of compounds **8**

Table S1: ^{13}C NMR data for compounds **1**, **3** ($CDCl_3$), **4**, **5** and **6a** (CD_3OD). The central spectral line of the solvent was used as a reference.

C	Vindoline 1	Mono-adduct 3	Di-adduct 4	Di-adduct 5	Gem adduct 6a
2	83.2	83.3	84.5	84.4	84.6
3	50.9	50.9	51.8	52.0	51.9
5	51.7	51.6	52.4	52.6	52.7
6	43.9	43.9	44.7	44.6	44.4
7	52.6	52.7	54.1	54.0	53.6
8	124.8	124.4	125.9	125.9	125.7
9	122.5	125.0	126.6	126.5	125.8
10	104.4	112.6	114.6	115.5	116.0
11	160.9	159.5	161.1	161.2	160.6
12	95.6	92.9	94.4	94.4	94.1
13	153.5	155.1	156.5	156.7	155.5
14	129.9	124.2	125.8	125.8	125.6
15	130.3	130.3	131.3	131.2	131.1
16	79.4	79.4	80.9	80.8	80.8
17	76.1	76.2	77.6	77.5	77.5
18	7.5	7.6	8.1	8.1	8.0
19	30.6	30.9	32.1	32.1	32.1
20	42.7	42.8	44.3	44.3	44.2
21	66.8	66.8	67.6	67.9	68.0
$\underline{C}OOCH_3$	171.7	171.8	173.4	173.4	173.4
$COO\underline{C}H_3$	52.0	52.3	52.9	52.9	52.9
$O\underline{C}OCH_3$	170.5	170.7	172.5	172.5	172.4
$OCO\underline{C}H_3$	20.9	21.6	20.8	20.8	20.8
$N\underline{C}H_3$	38.0	37.6	38.3	38.3	38.7
$ArO\underline{C}H_3$	55.1	55.8	56.4	56.6	56.6
1'		188.0	188.3	189.6	187.5
2'		145.1	146.7	148.7	145.2
3'		132.8	134.3	132.4	145.2
4'		186.5	188.3	187.8	187.5
5'		136.1	146.7	132.4	137.7
6'		137.0	134.3	148.7	137.7

Table S2. Energetics obtained at the DFT (M06-2X/6-311G**) level of theory, considering the methanol implicit solvent (CPCM). Values given in hartree.

	E	$H°_{0K}$	$H°_{298K}$	$G°_{298K}$
**6a1** anti1	-1072.4627	-1072.1481	-1072.1267	-1072.1974
**6a1** anti2	-1072.4626	-1072.1480	-1072.1263	-1072.1992
**6b** syn	-1072.4639	-1072.1490	-1072.1276	-1072.1980
TS anti1 ↔ anti2	-1072.4599	-1072.1455	-1072.1246	-1072.1953
TS anti1 ↔ syn	-1072.4300	-1072.1158	-1072.0951	-1072.1645
TS syn ↔ syn	-1072.4596	-1072.1451	-1072.1242	-1072.1242

I. Views of **6a** and **6b** isomers:

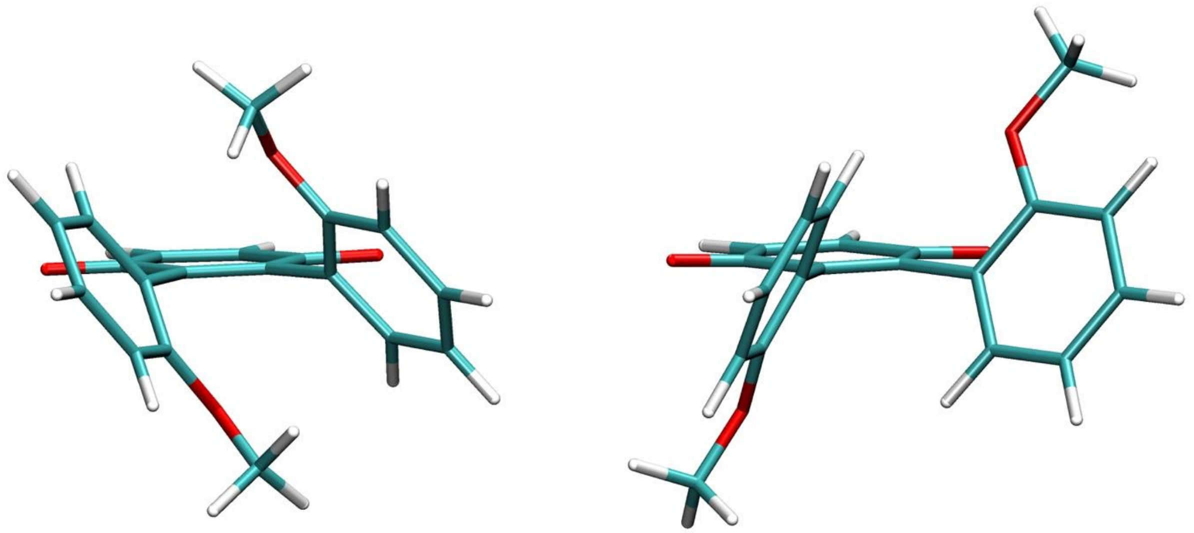

Fig. S1 6a1 anti1 and anti2 representations (from optimized M06-2X/6-311G** geometry).

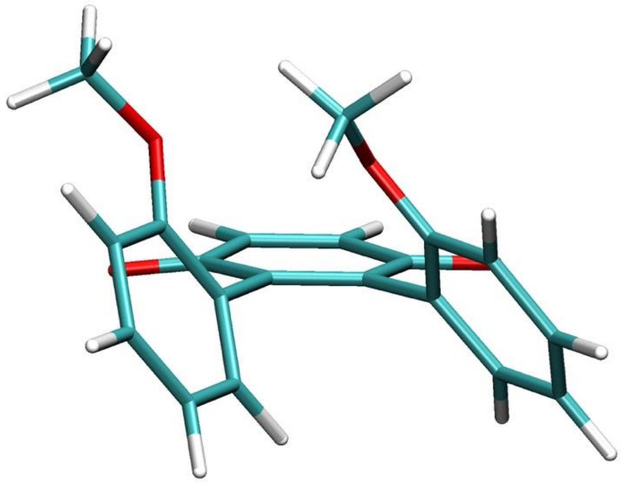

Fig. S2 6b syn representation (from optimized M06-2X/6-311G** geometry).
